# SARS-CoV-2 infection during pregnancy and necrotizing enterocolitis: case report and review of the literature

**DOI:** 10.1186/s13052-025-02155-9

**Published:** 2025-12-15

**Authors:** Gregorio Serra, Marco Pensabene, Deborah Bacile, Maria Rita Di Pace, Donatella Ferraro, Mario Giuffrè, Ettore Piro, Sergio Salerno, Ingrid Anne Mandy Schierz, Maria Sergio, Giovanni Corsello

**Affiliations:** 1 Neonatal Intensive Care Unit, Department of Health Promotion, Mother and Child Care, Internal Medicine and Medical Specialties “G. D’Alessandro”, University of Palermo, Palermo, Italy; 2 Paediatric Surgery Unit, Department of Health Promotion, Mother and Child Care, Internal Medicine and Medical Specialties “G. D’Alessandro”, University of Palermo, Palermo, Italy; 3 Clinical Microbiology Laboratory, Department of Health Promotion, Mother and Child Care, Internal Medicine and Medical Specialties “G. D’Alessandro”, University of Palermo, Palermo, Italy; 4 Paediatric Radiology Unit, Department of Health Promotion, Mother and Child Care, Internal Medicine and Medical Specialties “G. D’Alessandro”, University of Palermo, Palermo, Italy

**Keywords:** NEC, Newborns, SARS-CoV-2 infection, Gestation, COVID-19

## Abstract

**Background:**

Necrotizing enterocolitis (NEC) is a severe intestinal disease primarily affecting premature newborns, with high morbidity and mortality. Although typically linked to prematurity and low birth weight, recent reports have suggested a potential association between NEC and maternal SARS-CoV-2 infection during pregnancy. This study aims to explore this relationship through a systematic literature review and the presentation of a novel clinical case.

**Case presentation:**

A female neonate born at 37 + 2 weeks of gestation to a mother with third-trimester SARS-CoV-2 infection presented with feeding intolerance, abdominal distension, and bloody stool at 8 days of life. Diagnostic evaluation confirmed necrotizing enterocolitis, necessitating multiple surgeries for bowel resection, stoma creation, and repair of perforations. Despite intensive multidisciplinary care, the patient developed severe complications, including short bowel syndrome and intestinal failure, leading to death at 18 months. SARS-CoV-2 IgG positivity in the neonate suggested transplacental antibody transfer after maternal infection, while most of the other possible risk factors were excluded.

**Conclusions:**

The presented case highlights the potential role of maternal SARS-CoV-2 infection in necrotizing enterocolitis pathogenesis. Literature review identified 10 studies documenting similar associations. Cases ranged in severity, with outcomes influenced by gestational age, infection timing, and neonatal risk factors. The proposed mechanisms include virus-induced inflammation, placental dysfunction, and altered neonatal gut microbiota. Our findings emphasize the importance of considering maternal SARS-CoV-2 infection as a contributing factor in NEC, especially in term neonates without known predisposing conditions. Further research is crucial to elucidate the possible link between COVID-19 and NEC, and guide management of affected neonates. Preventive strategies, including vaccination and optimized prenatal care, are essential to mitigate risks.

## Background

Necrotizing enterocolitis (NEC) is a severe disease frequently occurring in preterm infants, and in those weighing at birth less than 1500 g. Incidence rates are variable, ranging from 2% to 13% [1, 2]. In preterm babies, it is associated with high mortality (20–30%), increasing up to 50% in cases of extremely low birth weight (ELBW) neonates undergoing surgical treatment [1, 3]. Although 90% of infants developing NEC are born prematurely, also full-term ones may develop the disease, especially in the presence of risk factors, such as congenital heart disease (CHD), fetal growth restriction (FGR), exchange transfusions, polycythemia, thrombotic conditions, sepsis and prenatal exposure to abuse substances [4, 5]. The etiology is multifactorial, and the pathogenic mechanisms involved include hypoxia, inadequate anti-microbial defenses due to immature Paneth cells and peristalsis, physiological immaturity of enterocytes tight junctions, mucosal inflammation linked to anemia (especially after red blood cell [RBC] transfusions) [6, 7]. Anemia, indeed, can impair splanchnic perfusion, resulting in tissue hypoxia, anaerobic metabolism, and accumulation of its by-products such as lactic acid. It can also impair the normal maturation of vascular autoregulation in the premature intestine, predisposing to ischemic injury, and possibly, NEC [8]. Furthermore, Prgomet et al. identified anemia as the major risk factor of gastrointestinal perforation (GIP), usually occurring in premature babies with NEC, as well as in small for gestational age (birth weight below 10th percentile) infants [9].

Main clinical manifestations of NEC are drowsiness, unstable body temperature, apnea, bradycardia, vomiting and high gastric residuals, abdominal distension, bloating and bloody stool. These signs and symptoms are often associated with increased inflammation indices and thrombocytopenia [10]. However, the hallmark diagnostic finding of NEC is pneumatosis intestinalis (result of intramural gas within the bowel wall, produced by bacterial fermentation within the gut lumen), revealed by imaging investigations (X-ray and ultrasound), which may be confirmed on gross examination and histopathology in those cases undergoing surgery. Other pathological pictures of the disease include portal venous gas, mucosal edema, epithelial sloughing/villous atrophy, secondary bacterial infiltration, vascular thrombosis, and discontinuous and variably deep coagulative necrotic intestinal segments, or “skin lesions” [11, 12]. Medical treatment consists of broad-spectrum antibiotics, fasting and parenteral nutrition [10, 13]. In cases complicated by intestinal perforation, surgery is required. Exploratory laparotomy with stoma creation is the most common approach. Nevertheless, consensus on this topic is lacking, as the varying clinical conditions of patients may influence the surgical strategy [14, 15]. In recent years, with the emergence of the SARS-CoV-2 pandemic, cases of NEC associated with maternal COVID-19 have been increasingly reported. The purpose of our review is to analyze all documented cases of NEC in neonates associated with SARS-CoV-2 infection occurring either in their mothers during pregnancy or after birth. The literature revision has been enriched by the personal contribution of an additional patient observed at the Mother and Child Department of the University of Palermo, Italy, between September and December 2022, to widen the scientific database. Our study has also the aim of promoting greater awareness among clinicians of the possible pathophysiological correlation between such diseases, which may have significant implications for neonatal health.

## Case presentation

**Fig. 1:**
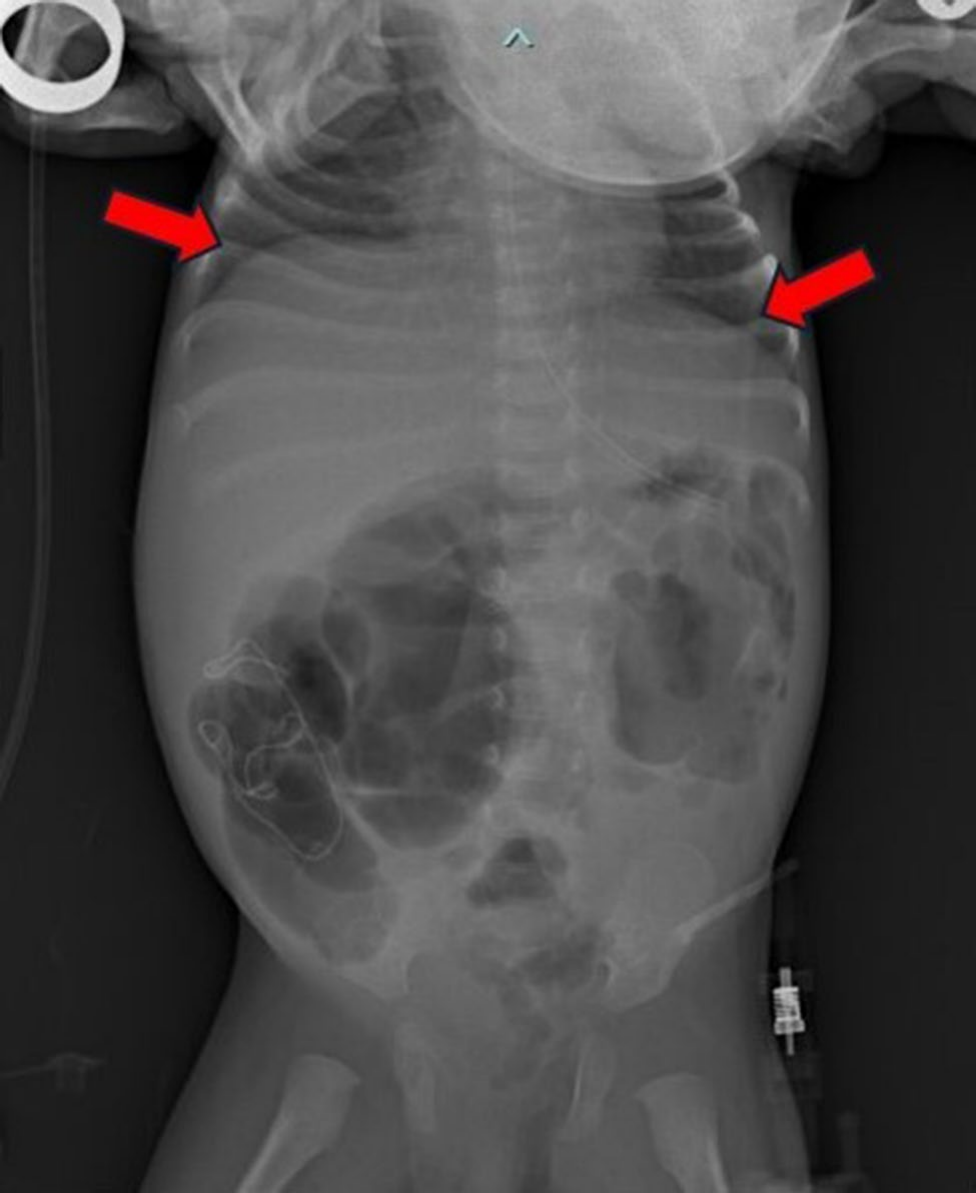
Pre-operative abdomen X-ray, showing a huge dilatation of ileal loops along with air bubbles below both hemidiaphragms (cupola sign, indicated by arrows)

**Fig. 2:**
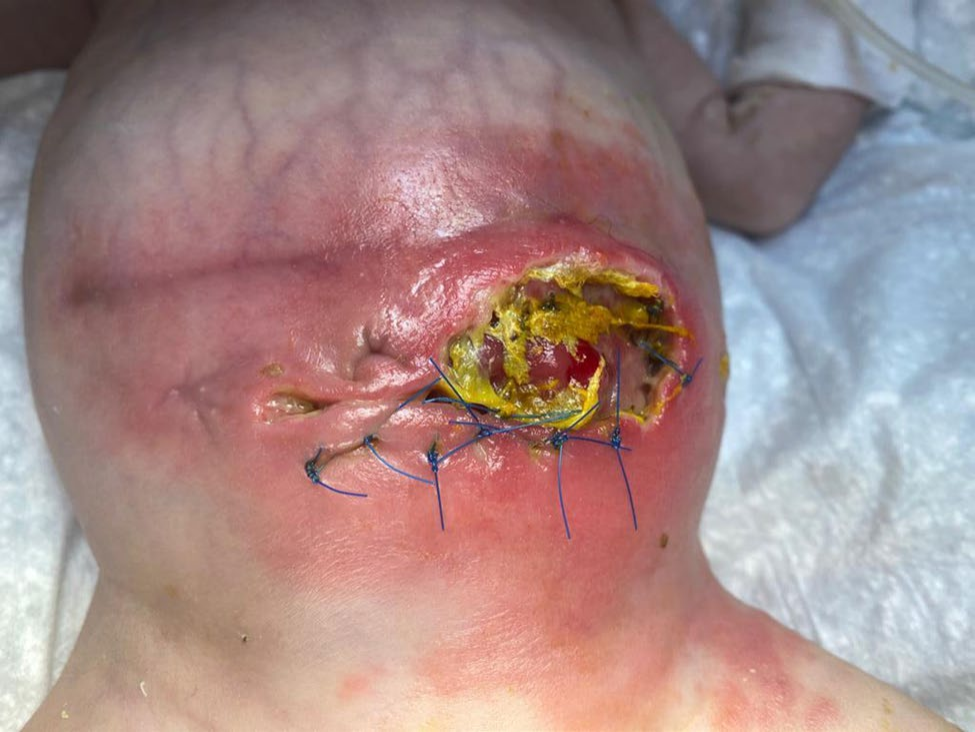
Extensive dehiscence involving the abdominal wall and the jejunum-ileum anastomosis

**Fig. 3:**
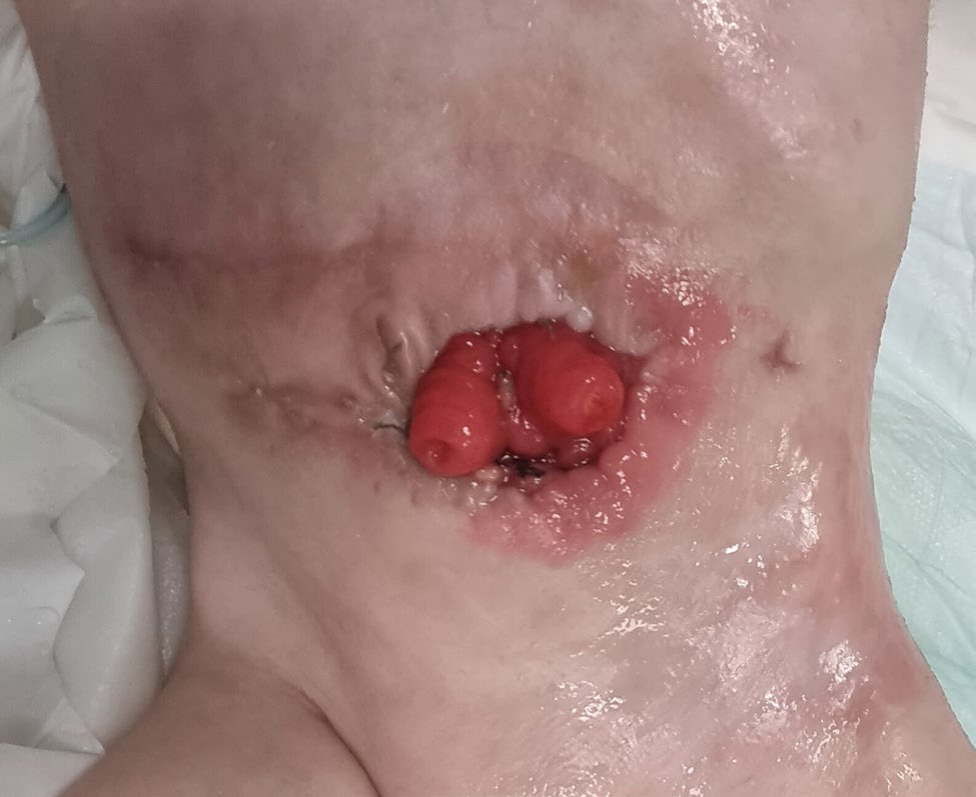
Jejunostomy, 20 days postoperatively after the last repair of multiple perforations in the distal ileum

A female neonate was born at 37^+ 2^ weeks of gestation (WG) by spontaneous vaginal delivery, to healthy, non-consanguineous parents. Family history was unremarkable. Prenatal ultrasound (US) evaluations documented FGR and abnormal blood flow, with a raised umbilical artery pulsatility index (>95th percentile), in the early third trimester. A complete serological profile for TORCH agents yielded negative results throughout the entire gestation, which was managed at the main public hospital of a small town in a neighboring province of Western Sicily, near our Department in Palermo, Italy. Pregnancy history, however, revealed a SARS-CoV-2 infection contracted during the third trimester. The woman had not been immunized during gestation due to the unavailability of a specific vaccine at that time. Symptoms were mild and flu-like (rhinorrhea, sore throat, cough, and fever), lasting approximately one week and not requiring hospitalization. The diagnosis was confirmed by a positive real-time PCR nasopharyngeal swab and by serological testing, which showed positive SARS-CoV-2–specific IgM (index >1). The cutoff value (index 1) was defined as the signal-to-cutoff (S/CO) ratio, according to the manufacturer’s instructions for the SARS-CoV-2 IgM chemiluminescent microparticle immunoassay (AdviseDx, Abbott^®^, Lake Forest, IL, USA) [16]. Other obstetric and/or pregestational pathological conditions (e.g., diabetes, hypertension, thrombophilia, autoinflammatory diseases, substance use) were ruled out through specific clinical and laboratory assessments (e.g., glycated hemoglobin [HbA1c], regular blood pressure monitoring, and urine drug screening for common substances of abuse). In particular, acquired thrombophilias—including lupus anticoagulant (LAC), anti-cardiolipin antibodies (IgG and IgM), and anti-β2 glycoprotein I antibodies (IgG and IgM)—were also investigated in the mother and tested negative. The adaptation to extrauterine life was regular, and Apgar scores were 8 and 10, respectively at 1 and 5 min. At birth, anthropometric measures were as follows: weight 1940 g (g) (1st centile, -2.31 standard deviations, SD), length 44 cm (3rd centile, -1.91 SD), occipitofrontal circumference (OFC) 32 cm (15th centile, 1.04 SD). Neither dysmorphic features nor neurological abnormalities were noticed at first clinical examination, or hereafter throughout the patient’s clinical evolution. Postnatally, at day 8 of life, enteral feeding intolerance and poor sucking along with abdominal distension, bilious vomiting and bloody stool, were observed. Owing to clinical suspicion of NEC, the patient underwent emergency laparotomy at the Pediatric Surgery Unit of the birthing center, within the same hospital where the mother had received prenatal care. Two main intestinal loops were frankly necrotic: the terminal ileal loop involving the ileocecal valve, and a more proximal ileal segment. The surgical procedure therefore consisted of resection of the necrotic bowel, with a primary ileocolic anastomosis and the creation of a proximal ileostomy. A few days after stoma closure, which was performed one month later, the patient’s clinical condition deteriorated due to fecal peritonitis and pneumoperitoneum. She was subsequently transferred to our Department of Mother and Child Care at the University of Palermo, where an emergency exploratory laparotomy was performed. The bowel appeared compromised, with enteric secretions in the peritoneal cavity and evidence of fecal peritonitis. Extensive adhesiolysis was performed in a cranio-caudal direction, allowing full exploration of the intestinal tract. Bowel exploration revealed partial dehiscence of the ileo-ileal anastomosis at the site of stoma closure. In addition, complete dehiscence of the ileocolic anastomosis was observed, with the anastomotic site being densely adherent to the duodenum and liver. A marked dilatation of the ileal loop proximal to the ileocolic anastomosis was noted, while the colon appeared empty. Resection of the most severely damaged intestinal segments was therefore performed, followed by new primary end-to-end ileocolic and ileo-ileal anastomoses. A protective stoma was fashioned in the distal jejunum. Histological examination showed mucosal atrophy of the small intestine with associated hemorrhagic infarction of the lamina propria, necrosis, edema, vascular congestion of the submucosa, and a moderate lymphoplasmacytic and neutrophilic infiltrate, consistent with necrotizing enterocolitis (NEC). One month later, after an uneventful postoperative course, the jejunostomy was closed. However, on postoperative day 4, the patient developed clinical deterioration due to intestinal obstruction, with radiological evidence of peritonitis and pneumoperitoneum (Fig. 1), prompting a new exploratory laparotomy. Extensive bowel exploration was not safely feasible due to multiple dense adhesions. A spontaneous ileal perforation was found distal to the previously closed stoma, which appeared to be healing normally. The perforation was repaired, and peritoneal drainage was left in situ. Three days later, the patient experienced evisceration due to extensive dehiscence involving the abdominal wall and the jejuno-ileal anastomosis (Fig. 2). A further emergency laparotomy revealed multiple spontaneous perforations in the distal ileal loop. A new stoma was created in the healthy jejunum, proximal to the area affected by the perforations, which were repaired (Fig. 3). At that time, further bowel exploration was not possible due to the patient’s critical condition. The clinical course was subsequently complicated by anemia and coagulopathy, requiring transfusion of red blood cells and plasma. After stabilization, completion of the diagnostic work-up was possible. It initially included an assessment of hereditary thrombophilias, such as *MTHFR* mutations (C677T and A1298C), which were not detected, as well as measurement of anticoagulant proteins (Protein C, Protein S, and Antithrombin III), all within the reference ranges. At this time, serological screening for TORCH agents was completed, resulting negative. Syphilis testing and urinary detection of CMV DNA, indeed, had already been performed in the first days of life before the initial surgery, both yielding negative results. The only positive finding was the presence of SARS-CoV-2 specific IgG antibodies (>50 AU/ml, AdviseDx SARS-CoV-2 IgG II assay, Abbott^®^, Sligo, Ireland) [16], consistent with transplacental transfer due to maternal infection, as previously described. Blood and urine cultures were also obtained, ruling out systemic bacterial or fungal infections. Additional PCR testing for major bacterial pathogens (e.g., Salmonella, Shigella, Escherichia coli, Clostridium difficile) and viruses (including enterovirus, adenovirus, and rotavirus) was performed on nasopharyngeal and rectal swabs, as well as stool samples, all yielding negative results. Furthermore, PCR testing for SARS-CoV-2 genome in biopsy samples of colon and ileum was conducted and showed negative findings (see details below in the following subparagraph). An extended metabolic screening was carried out and periodically repeated according to the protocol of the local Reference Center (Western Sicily) for Congenital Metabolic Disorders, all with negative results. Abdominal Doppler ultrasonography provided valuable information on intestinal perfusion and revealed no signs of ischemia. Echocardiography ruled out underlying congenital heart disease that could lead to hemodynamic instability potentially responsible for NEC. Moreover, head ultrasonography and sensory screenings did not detect any congenital malformations or defects; therefore, molecular cytogenetic or next-generation sequencing analyses were not performed, also considering the absence of dysmorphic features and family or NEC recurrence history.

During the first two months of life, parenteral nutrition was administered to support the low enteral feeding provided (approximately 35% of total fluid intake), mainly through a nasogastric tube. Initially, breast milk was given, followed by an amino acid–based formula due to both poor tolerance to enteral feeding (i.e., gastric distension) and significant fluid loss (an average of about 95 ml/kg/day, compared to 150 ml/kg/day administered) from the proximal jejunal stoma. At 3 months of age, she underwent surgery for stoma closure and end-to-end intestinal anastomosis. Two months later, a mild increase in enteral feeding was achieved (40% of total nutritional requirements provided by the amino acid formula), accompanied by normal stool output and no regurgitation, vomiting, or signs suggestive of digestive tract obstruction. Nonetheless, suboptimal parenteral nutrition (PN), administered only through midline venous catheters—with a reduced intake of macronutrients (i.e., 1 g/kg/day of amino acids and 1.1 g/kg/day of lipids) to avoid a high osmolar load—due to progressive venous depletion, her dystrophic state, and the parents’ refusal of central line catheter placement, led to severe global growth delay. This included weight (2970 g, < 0.4th percentile, − 6.531 SD), length (57 cm, < 0.4th percentile, − 2.680 SD), and OFC (37.1 cm, < 0.4th percentile, − 3.005 SD), according to the World Health Organization growth charts for neonatal and infant close monitoring [17]. Therefore, she was transferred to a Pediatric Gastroenterology and Nutrition Reference Centre in Central Italy to continue complex multidisciplinary management, including an intestinal rehabilitation program. Initially, this yielded beneficial results; however, subsequent complications—including dyselectrolytemia, dysprotidemia, recurrent infections, and metabolic acidosis—arising from persistent intestinal failure, hindered bowel transplantation and led to early death at 18 months of age.

### Detection of SARS-CoV-2 genome in colon and ileum biopsy samples

Biopsy samples of the colon and ileum, embedded in paraffin, were treated overnight at 56 °C with 200 µL of Feoli-Fonseca reagent and Proteinase K at a concentration of 300 µg/mL. Following Proteinase K denaturation and centrifugation for 5 min at 13,000 rpm, the supernatant was used for SARS-CoV-2 genome detection using two diagnostic real-time PCR kits.

Viral genome amplification – real-time PCR

1. Xpert^®^ Xpress SARS-CoV-2 Plus (Cepheid, Sunnyvale, California, USA): 300 µL of the supernatant were loaded into the dedicated cartridge. The system performed automated nucleic acid extraction and amplification by real-time PCR.

Target genes: N2, E, RdRp

2. Allplex™ SARS-CoV-2 Assay (Arrow Diagnostics, Seegene Inc., South Korea): nucleic acid extraction was performed using the QIAamp DNA Mini Kit (Qiagen, Hilden, Germany) according to the manufacturer’s instructions. Elution was performed in 100 µL of water, and 5 µL of the eluate were used for real-time PCR.

Target genes: E, N, S, RdRp

The entire procedure was performed in triplicate for both colon and ileum samples. SARS-CoV-2 genome was not detected in either tissue sample using either of the two amplification methods.

## Discussion and conclusions

Necrotizing enterocolitis (NEC) is an inflammatory bowel necrosis typically affecting premature newborns, among which it represents one of the major causes of mortality. Its etiopathogenesis is complex: the imbalance between pro- and anti-inflammation cytokines seems to be the main pathogenetic pathway, together with the failure to regenerate a normal mucosal enteric barrier. Increased activity of nitric-oxide and platelet activating factor (PAF), have also been advocated among the pathogenetic mechanisms, although their involvement is not fully elucidated. Moreover, several studies reported that genetic variants, leading to upregulation of downstream signaling receptors of Toll-like receptor-4 (TLR-4), can increase the risk of NEC [18]. Lastly, recent literature reports highlighted how the initial intestinal colonization of premature infants may be strongly affected by obstetric diseases or abnormalities [19, 20]. The most common significant prenatal risk factors of NEC, indeed, include intrauterine hypoxia and/or impaired fetal perfusion for placental insufficiency (associated or not with increased oxidative stress [5, 21], documented in pregnant women with gestational diabetes mellitus), leading to fetal growth restriction and low birth weight, both present in the current patient.

Surgical intervention, to resect portions of the ischemic bowel, is required if the disease progresses to the advanced stages [22], in which intestine (specifically distal ileum and proximal colon) is susceptible to inflammatory processes and perforation, leading to peritonitis and pneumoperitoneum [23], both occurred in our baby. Stoma should be created in a healthy loop, proximal to the involved intestinal segment, and bowel resection of necrotic gut is mandatory. If extensive necrosis is found on intestinal exploration (NEC totalis), clinical management can be challenging: extensive resections should not be performed due to the risk of “short bowel syndrome”; however, leaving severely injured loops in place may lead to a deterioration in the clinical course [24]. Therefore, in these cases, extensive resection is avoided, and a “second look” surgery is commonly performed after 48–72 h, allowing a partial intestinal restoration [25]. Intestinal functional prognosis, indeed, is a key-point in the survival of NEC patients. In fact, it is the main factor responsible for Short Bowel Syndrome (SBS), which may lead in turn to serious late consequences including Intestinal Failure (IF), as evidenced also in our report. IF is defined as the need of parenteral nutrition (PN) for >90 days after gut resection, or as a bowel length of less than 25% of expected, but it has been found also in cases of medical NEC, that did not require intestinal resections [26]. However, the anatomic site of intestinal resection may represent a significant variable, as patients with large ileum resections, as in the present case, have shown a poorer intestinal prognosis if compared to those undergoing jejunal resection [27]– [28]. Despite advances in parenteral nutrition and intestinal rehabilitation, allowing good survival rates also in patients with < 10 cm left bowel, mortality still ranges between 20 and 30%, mainly due to prematurity and IF, with the latter being also the cause of the fatal outcome in our infant. It can reach 50% in patients undergoing surgical treatment, reflecting the worse status of disease in this specific population. An efficacious and customized parenteral nutrition, along with early refeeding with human milk play a key role in the favorable evolution of these patients [29–31], but none of these measures were available in the current case.

Other conditions different than prematurity and FGR, as for example congenital infections sustained by TORCH agents, or more recently also SARS-CoV-2, have been found to be responsible for intestinal inflammation, and then for variations of the microbiome. SARS-CoV-2 viral RNAs, more precisely, have been detected in placentas [32] and amniotic fluid of infected pregnant women (in our case the histopathological investigation was not performed due to the unavailability of the placental sample), and there they may be ingested by the fetus reaching the intestine already *in utero* [33]. COVID-19 has been, actually, implicated in the development of NEC in newborns, also if contracted antenatally by the mother, probably due to its role in contributing to intestinal inflammation [34].

**Fig. 4:**
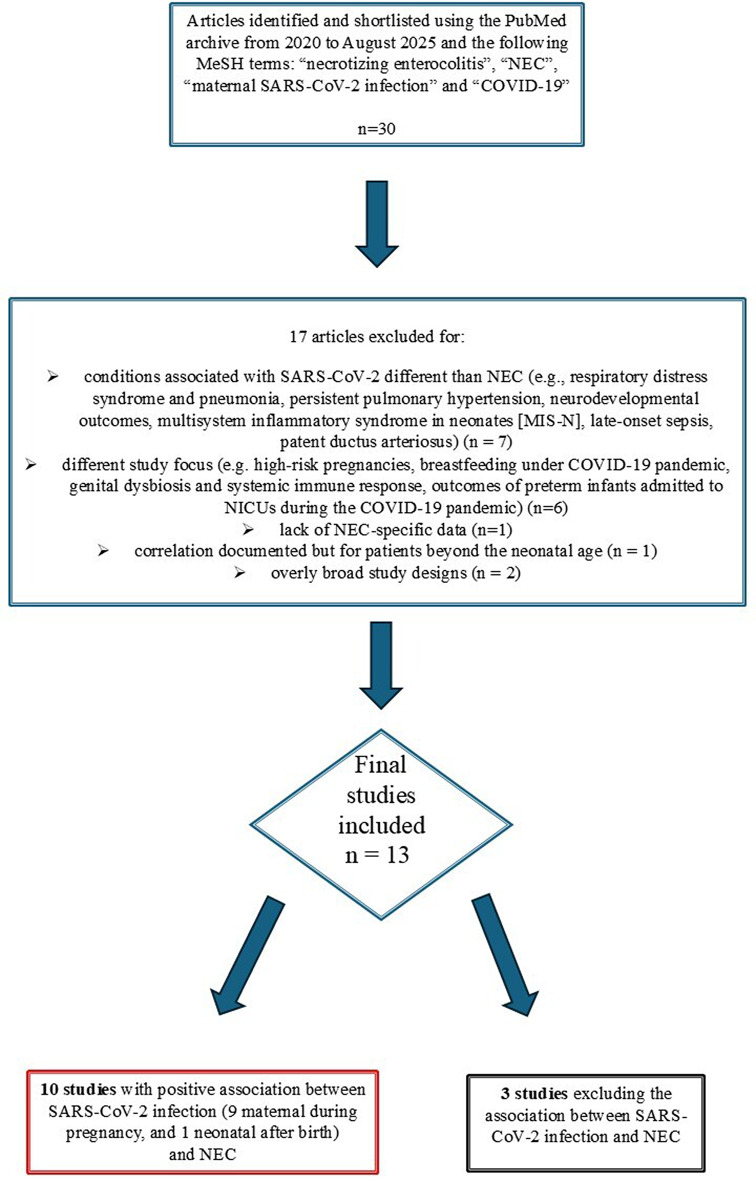
Flow chart representation of the design of the current review

Since the presented patient had no known risk factors for NEC and no other diagnostic findings—apart from specific SARS-CoV-2 IgG antibodies and a case of fetal growth restriction (FGR) temporally associated with prenatal maternal COVID-19—we conducted a systematic review of the literature. Publications were selected from the PubMed database between January 2020 and August 2025, aiming to identify studies describing the association between maternal SARS-CoV-2 infection during pregnancy (and/or after birth) and the development of necrotizing enterocolitis (NEC) in the newborn. For the purposes of the revision, the following key words were inserted alone or in combination into the search engines: “necrotizing enterocolitis”, “NEC”, “maternal SARS-CoV-2 infection”, and “COVID-19”. After performing such research, we found 30 papers. Most (*n* = 7) were irrelevant, due to the description of conditions associated with SARS-CoV-2 different than NEC (e.g., respiratory distress syndrome and pneumonia, persistent pulmonary hypertension, neurodevelopmental outcomes, multisystem inflammatory syndrome in neonates [MIS-N], late-onset sepsis, patent ductus arteriosus), and were then excluded. Others do not meet the inclusion criteria focused on the association between SARS-CoV-2 infection (maternal or neonatal) and necrotizing enterocolitis (NEC) for other reasons: different study focus (e.g. high-risk pregnancies, breastfeeding under COVID-19 pandemic, genital dysbiosis and systemic immune response due to SARS-CoV-2 infection, outcomes of preterm infants admitted to NICUs during the COVID-19 pandemic) (*n* = 6), lack of NEC-specific data (*n* = 2), documented correlation for patients beyond the neonatal age (*n* = 1), or overly broad study designs (*n* = 2). Finally, we collected and analyzed 13 studies describing newborn patients, in which an association between SARS-CoV-2 infection and NEC has been identified (confirmed in 10 of them, excluded in 3, Fig. 4). In addition, we personally contributed to widen the literature data, reporting our newborn with maternal SARS-CoV-2 infection during pregnancy, and subsequently developing a severe form of NEC. This required a particularly complex multidisciplinary management, due to postsurgical complications which led to short bowel syndrome, intestinal failure and finally to death. We compared our case to those described in previous studies, highlighting risk factors, clinical features, diagnostic methods and outcomes, to provide an updated overview on the existing literature regarding the relationship between COVID-19 and NEC (Table 1).

**Table 1:** Comparison between our patient and the previously reported ones showing a positive association between SARS-CoV-2 infection during pregnancy or after birth and NEC

Authors	Time of maternal SARS-CoV-2 infection during pregnancy	Gestational age	Concurrent risk factors	Time at NEC diagnosis	Surgery	Modalities of SARS-CoV-2 detection in the newborn	Outcome
Liu et al. (2020) [34]	Third trimester	35^+ 5^ WG	Prematurity	Third week after birth	NR	Positive pharingeal SARS-CoV-2 RT-PCR test	NR
Mannix MK et al. (2021) [35]	Upon admission to the labor and delivery unit	34	Prematurity	At approximately 30 h of life	Surgical intervention not required.	Positive nasal SARS-CoV-2 RT-PCR test	Resolution of NEC after 10 days of antibiotic therapy
Vizheh M et al. (2021) [36]	Perinatal (not well specified)	Preterm (not specified)	Prematurity	NR	NR	Positive nasal SARS-CoV-2 RT-PCR test	Death in neonatal age (not specified)
Rajurkar R et al. (2021) [37]	After birth	1 female term baby and 1 male preterm (36^+ 4^ WG)	No risk factors for the female term baby; prematurity, low birth weight, twin pregnancy, SGA, dysmorphisms and congenital heart disease for the male neonate	6 days and 18 days respectively	Not required in the first case, not possible due to severe and instable conditions in the second	Positive nasal SARS-CoV-2 RT-PCR test for both	The first newborn discharged after 7 days after bowel rest and iv broad-spectrum antibiotics; death in neonatal age for the second patient despite aggressive management (steroids, IVIg, remdesivir, inotropes, ventilatory support)
Gupta K et al. (2022) [38]	First trimester	34^+ 5^ WG	Prematurity, low birth weight (2200 g)	26 h of life	Emergency laparatomy performed at 28 h of life: large gastric perforation and extensive necrosis involving terminal ileum and entire colon. Stomach perforation was closed and loop ileostomy was done	High titers of total COVID antibodies in the mother and infant (1350 and 725, respectively)	Severe metabolic acidosis and resistant shock requiring multiple inotropes. Baby gradually improved, minimal feeds were started on 10th postoperative day and finally discharged on day 19 of life
Nita R et al. (2022) [39]	38–39 weeks of gestation	38–39	-	6 days	25 cm of resected ileum	SARS-CoV-2 IgM+/IgG+ Negative nasal SARS-CoV-2 RT-PCR test	NR
Angelika D et al. (2022) [40]	NR	< 34 WG (in 4 patients) 34–36 WG (in 1 newborn)	Low-birth-weight < 2500 g in all 5 patients (2 of them < 1500 g); asphyxia (in 4) and caesarean section (in 4)	NR	NR	Positive SARS-CoV-2 naso-pharyngeal RT-PCR in 2 newborns, negative in 3 (in these latter the same test was found positive in the mother during gestation)	Death in 4 cases, survival in 1
Jin CJ et al. (2023) [41]	15 weeks of gestation	27^+ 4^	Extreme prematurity	26 days (31 weeks of corrected age)	Jejunal and ileal resections	Increased levels of neonatal stool spike protein	NR
Jamali Z et al. (2023) [42]	At hospital admission prior to delivery	Full-term	-	6 days	NR	Positive nasal SARS-CoV-2 RT-PCR test	Discharged after 29 days and followed for eight months (persistent mild hydrocephalus)
Sidatt M et al. (2023) [43]	Maternal COVID infection confirmed approximately 20 days after birth	38	-	27 days	The gut abnormalities started approximately from 50 cm after the duodenal flexure till the ileocecal valve	Positive nasal SARS-CoV-2 RT-PCR test	Death within a few hours after surgery
Our patient	Third trimester	37^+ 2^	FGR (raised umbilical artery pulsatility index (> 95th centile); low birth weight	8 days	Ileo-colic resection and anastomosis, proximal ileostomy (day 8). Dehiscence of ileocolic and ileo-ileal anastomoses: resection of intestinal segments, distal jejunostomy (1 month and 15 days). Repairs of perforations in the distal ileum. New stoma in the healthy proximal jejunum (1 month and 19 days, and 1 month and 22 days).Stoma closure and end-to-end intestinal anastomosis (3 months)	SARS-CoV-2 IgG+ Negative nasal SARS-CoV-2 RT-PCR test	*Exitus* at 18 months of age

IV = immunoglobulins; NR = not reported; RT-PCR = real time polymerase chain reaction; WG = weeks of gestation

In all described cases, the diagnosis was made through molecular detection on nasopharyngeal swabs and/or serological tests. Only in the patient reported by Jin et al. [35]. was the viral genome detected in stool samples. Therefore, the negative results found in the colonic and ileal biopsy samples in the present analysis do not exclude a potential pathogenic role of SARS-CoV-2 during pregnancy in our case, particularly regarding intestinal injury. As for the severity of the outcomes, we observed—although information on clinical progression was not available for all patients included in the review—a more extensive bowel involvement in the extremely preterm newborn described by Jin et al. [35], whose mother was infected during the second trimester of pregnancy. Similarly, a case of gastric perforation was reported by Gupta et al. in a patient born to a mother who contracted the disease during the first trimester of gestation [36]. An even more unfavorable course—ultimately resulting in a fatal outcome—was observed in the second of the two patients reported by Rajurkar et al. [37], who was a twin late preterm, small for gestational age, and additionally affected by a likely concomitant genetic condition including a congenital heart disease. Conversely, less marked intestinal damage was observed in the patient reported by Nita et al. [38], who underwent only ileal resection, and even milder involvement was reported in those described by Mannix et al. [39], in whom conservative treatment with antibiotic therapy led to complete recovery, and in the case reported by Jamali et al. [40], who was discharged at around one month of age and was followed over time only due to the co-occurrence of mild hydrocephalus. As expected, in the newborns reported by the aforementioned Authors [38–40], as well as in the patient described by Sidatt et al. [41]. and in the first of the two neonates reported by Rajurkar et al. [37], the infection occurred during the third trimester, around the time of delivery, or even after birth. Angelika et al. [42], in an observational study conducted on 125 subjects, found a relationship with NEC in neonates born to SARS-CoV-2-positive mothers. In such newborns, the most significant associated risk conditions included positive SARS-CoV-2 nasopharyngeal RT-PCR results (as in our experience, in this study most of the neonates [60% of the 5 who developed NEC] had negative RT-PCR results), asphyxia, lower gestational age, and lower birth weight, which were related to increased mortality rates (only one of those developing NEC survived). Vizheh M et al. as well, conducted in Iran in 2021 a cohort study on 255 neonates born to mothers with COVID-19 infection to investigate their clinical characteristics and outcomes [43]. These Authors observed that one of the 6 deaths documented in their cohort occurred in a premature girl newborn with a positive SARS result affected by necrotizing enterocolitis (NEC). Finally, the study of Jamali et al. explicitly identifies necrotizing enterocolitis (NEC) as part of the clinical spectrum of multisystem inflammatory syndrome in the newborn (MIS-N). The authors of this report emphasize that maternal SARS-CoV-2 infection may be associated with a neonatal multisystem inflammatory response, in which NEC can represent one of the clinical manifestations [40]. Despite the above-mentioned studies, current evidence on the topic remains limited, with only a few case reports, one cohort study, and a single observational study (the most comprehensive investigation published to date on this argument) [42] suggesting a potential association between SARS-CoV-2 infection—either maternal during pregnancy or neonatal—and the development of necrotizing enterocolitis (NEC). Conversely, a case-control study of Heyward EB et al. [44]. did not find a significant increase in the incidence of NEC among neonates born to SARS-CoV-2 positive mothers, highlighting the need for cautious interpretation. Similarly, a prospective, multi-institutional observational study [45] analyzed data on pregnancy outcomes, neonatal health, and congenital anomalies—including gastrointestinal conditions such as necrotizing enterocolitis—in newborns of women who contracted COVID-19 during gestation, comparing findings from before and after the onset of the pandemic. This analysis revealed no statistically significant differences in rates of necrotizing enterocolitis or gastroschisis. Furthermore, a comparative cohort study conducted in Sweden in 2024 [46], examining preterm infants (< 35 weeks of gestation) during the pre-pandemic and pandemic periods, demonstrated a slightly lower incidence of necrotizing enterocolitis (NEC) during the pandemic. These findings seem to suggest no direct association between COVID-19 and an increased population-level incidence of NEC. Therefore, the available data are not entirely consistent (Fig. 4). Although the overall current evidence from the literature points toward a possible link between NEC and SARS-CoV-2 infection either gestational or in the newborn, they are not yet sufficient to establish a causal relationship. To better understand any potential connection, larger-scale studies with prospective designs are warranted. In our newborn, although it might be hypothesized that the disease developed independently of the viral infection, however we raised the concern that maternal COVID-19 disease could have exerted a determining pathogenic role. This plausible correlation may be explained both by the strict temporal correlation with the flowmetric abnormalities revealed on obstetric doppler ultrasound evaluations, and mainly by the absence of the other possible pathological conditions investigated. Actually, they were assessed through comprehensive clinical assessment, as well as laboratory (e.g., coagulation and microbiological tests) and imaging investigations (heart and abdominal Doppler US to rule out congenital cardiopathy and intestinal ischemia, respectively), but were deemed not plausible due to negative findings. To further strengthen the suggested harmful effect of SARS-CoV-2 on bowel, we also know today the progressive decrease, until the current lack (year 2025), of published studies on the correlation between COVID-19 and NEC. Such downward trend may be likely linked with the mutations the virus underwent from the beginning of the pandemic, leading to variants with lower virulence (i.e., omicron), as well as to the development of vaccines against SARS-CoV-2, similarly to what has been observed for other serious clinical pictures associated with the novel coronavirus infection like Multisystem Inflammatory Syndrome in Children (MIS-C) and/or Neonates (MIS-N), whose incidence dramatically dropped down in the last months. The pathophysiological mechanisms involved in NEC enclose, among the others, mucosal inflammation, which may be linked with the detrimental effect of various pathogens including SARS-CoV-2. Some pathogenetic hypotheses have been proposed and, in particular, abnormal immunological reactivities in pregnant women with COVID-19 may present potential additional risks to the fetus, as maternal autoantibodies unleashed by SARS-CoV-2 infection which may attack fetal cells. Despite the documented evidence in neonates of the association between necrotizing enterocolitis and prenatal COVID-19, as observed also in our patient, the precise molecular pathways linking the diseases are not fully understood [47, 48]. Further studies are required to explore such correlation in both preterm and full-term newborns, and to better understand the mechanisms of *in utero* transmission and damage caused by the virus to the fetus and newborn. In this perspective, the present study adds significant findings to the few descriptions of the literature. The peculiar contribution of our experience is given by the specific clinical profile of our patient, characterized by the lack of concurrent risk factors (the FGR is raised just after the occurrence of maternal infection) potentially contributing to the disease. The information provided may help neonatologists remain aware of the potential co-occurrence of maternal SARS-CoV-2 infection and necrotizing enterocolitis (NEC), as well as of its possible complications—particularly in high-risk cases. These include neonates with fetal growth restriction (FGR) and low birth weight, as in the present case, whose clinical course may be affected by underlying or associated conditions [37, 42, 49–52]. These factors can contribute to increased disease severity and potentially life-threatening outcomes, requiring a careful, multidisciplinary approach involving neonatologists, obstetricians, pediatric surgeons, radiologists, microbiologists, and pathologists. Given the complexity of management, preventive strategies may play a pivotal role and should be actively promoted. These should focus on infectious disease prophylaxis—particularly vaccination (including SARS-CoV-2, which was unavailable at the time of our proband’s gestation)—in women planning pregnancy. In addition, optimizing maternal glycometabolic status and appropriately managing obstetric or pregestational risk factors (e.g., hypertension, thrombophilia) are essential. Together, these interventions may reduce NEC incidence and its adverse outcomes, ultimately improving the life expectancy and quality of life of affected infants and their families.

In conclusion, necrotizing enterocolitis is a potentially life-threatening condition whose evolution may depend on multiple predisposing and associated factors [37, 42, 49–52]. Our review highlights the possible role of prenatal SARS-CoV-2 infection in the pathogenesis of NEC, alongside well-established risk factors such as FGR, low birth weight, and other prenatal infections. Neonatologists should be aware of these associations and ensure high-risk cases receive multidisciplinary and integrated care.

## Data Availability

The datasets used and analyzed during the current study are available from the corresponding author on reasonable request.

## Abbreviations

CHD: Congenital heart disease
FGR: Fetal growth restriction
GIP: Gastrointestinal perforation
IF: Intestinal Failure
MIS-C: Multisystem Inflammatory Syndrome in Children
MIS-N: Multisystem Inflammatory Syndrome in Neonates
NEC: Necrotizing enterocolitis
PI: Pulsatility index
PN: Parenteral nutrition
SBS: Short bowel syndrome
SD: Standard deviations
US: Ultrasonography
